# Robot-Assisted Extravesical Ureteral Reimplantation (REVUR) in Pediatric Patients: A New Standard of Treatment for Patients with VUR—A Narrative Review

**DOI:** 10.3390/children11091117

**Published:** 2024-09-12

**Authors:** Ciro Esposito, Claudia Di Mento, Mariapina Cerulo, Fulvia Del Conte, Francesco Tedesco, Vincenzo Coppola, Annalisa Chiodi, Giorgia Esposito, Leonardo Continisio, Marco Castagnetti, Maria Escolino

**Affiliations:** 1 Pediatric Surgery Unit, Federico II University of Naples, 80131 Naples, Italy; ciroespo@unina.it (C.E.); maria.escolino@unina.it (M.E.); 2 Internal Medicine Unit, Federico II University of Naples, 80131 Naples, Italy; 3 CEINGE Advanced Biotechnologies, 80131 Naples, Italy; 4 Pediatric Urology Unit, Pediatric Hospital Bambino Gesù, 00165 Rome, Italy

**Keywords:** REVUR, children, vesicoureteral reflux, robotic surgery, pediatric minimally invasive surgery

## Abstract

Robot-assisted extravesical ureteral reimplantation (REVUR) was described for the first time in 2004. Since then, the surgical approach of vesicoureteral reflux (VUR) has changed dramatically. The benefits of this technique are great when compared to the laparoscopic or traditional open approaches. A literature search of PubMed was performed to identify articles covering any aspect of REVUR in the pediatric population. A total of 108 papers published over the period 2004–2024 were collected. Of these, 40 studies were considered valuable in terms of obtaining a complete overview of the REVUR technique. This review aimed to describe the current state of the art of REVUR and define it as the new standard technique for surgical management of selected patients with VUR.

## 1. Introduction

Robot-assisted extravesical ureteral reimplantation (REVUR) was introduced over 20 years ago [1]. Since its introduction, it has gained favor among many pediatric surgeons and urologists due to its relative ease of execution compared to purely laparoscopic extravesical ureteral reimplantation (LEVUR). Furthermore, compared to open ureteral reimplantation (OUR), it offers better outcomes in terms of postoperative pain and morbidity.

The relevance of robotic ureteral reimplantation, compared to the traditional open and laparoscopic approaches, in children with vesicoureteral reflux (VUR), is primarily based on several key factors that can be summarized as follows:

- Minimally invasive surgery (MIS)—robotic reimplantation is less invasive than open surgery, which helps to enhance recovery after the operation;
- Enhanced accuracy—robotic surgery provides greater precision and control than standard laparoscopy, enabling a more accurate and delicate reconstruction of the ureterovesical anastomosis;
- Shorter hospitalization—it has been well demonstrated that minimally invasive approaches can provide shorter hospitalization stays compared to traditional approaches;
- Lower risk of complications—complications such as bleeding and infections are reduced compared to the open approach;
- Shorter recovery time—children undergoing robotic surgery can experience faster recovery compared to open approach and daily activities can be resumed earlier;
- Improved surgeon comfort—robotic systems can reduce surgeon fatigue and provide better ergonomics during surgery, leading to better surgical outcomes.

After a brief opening, this study aimed to review the current state of the art of REVUR as a new standard of treatment in selected pediatric patients and provide an updated overview of this topic. This review will not cover the detailed aspects of the endoscopic, open, and laparoscopic surgical approaches.

### 1.1. Vesicoureteral Reflux (VUR)

Vesicoureteral reflux (VUR) is among the most prevalent urological conditions in children, affecting around 1% of the pediatric population [2]. VUR is defined as a permanent or intermittent abnormal retrograde passage of urine from the bladder into the upper urinary tract due to a defective ureterovesical junction. Even if there is not a definitive algorithm for operative intervention in terms of type of surgery and time, the goals of its management include the prevention of recurrent urinary tract infections (UTIs), which can lead to kidney scarring, and avoiding long-term antibiotic therapy [2].

### 1.2. Preoperative Management

Before surgery, the patient should undergo a comprehensive medical history and physical assessment. Comorbidities and contraindications to pneumoperitoneum must be recognized. Preoperative investigations should include urinalysis and culture, serum creatinine, renal bladder ultrasound, a renal scan to analyze renal function in each patient and a voiding cystourethrogram (VCUG) [3]. It is mandatory to know the exact anatomy of every single patient in order to avoid surprises during the surgical procedure. Bowel preparation is usually recommended to help increase the limited intra-abdominal space.

### 1.3. Indications

Ureteral reimplantation is indicated in children with primary vesicoureteral reflux under the following conditions [3]:

- High grade (IV/V) reflux that persists in children beyond two or three years of age;
- The deterioration of renal function;
- A failure of medical management, evidenced by breakthrough infections;
- Significant adverse effects from continuous antibiotic prophylaxis;
- The inability to adhere to a long-term medical treatment plan;
- Parental preference [4].

The EAU guidelines [3] recommend considering surgical correction for patients with persistent high-grade reflux (grades IV/V). There is no clear agreement on the timing and type of surgical intervention. Reimplantation tends to yield better outcomes than endoscopic correction for the higher grades of reflux, while endoscopic injection can be effective for the lower grades [3]. Furthermore, management decisions are influenced by factors such as the presence of renal scars, clinical history, reflux grade, ipsilateral renal function, bilaterality, bladder function, associated urinary tract anomalies, age, compliance, and parental preference [4]. Febrile UTIs, high-grade reflux, bilaterality, and cortical abnormalities are recognized as risk factors for potential renal damage. Additionally, the presence of lower urinary tract dysfunction (LUTD) increases the risk of new scars [3].

### 1.4. Surgical Treatment

The literature outlines several common treatment options for vesicoureteral reflux (VUR), including observation (with or without antibiotic prophylaxis), endoscopic injection (EI), and ureteral reimplantation. Traditionally, OUR was considered the “gold standard” approach [5]. However, with advancements in techniques and technology, minimally invasive treatment options started to be available such as endoscopic injection (EI) of bulking agent and laparoscopic and robot-assisted management. The choice of surgical approach is ultimately based on patient-specific factors (age, weight, previous surgery) and surgeon preference. The surgical intervention restores the anatomical integrity of the ureterovesical junction affected by reflux.

### 1.5. Transperitoneal Extravesical Technique

The extravesical robot assisted laparoscopic ureteral reimplantation is performed following the same steps as the open Lich-Gregoir technique. The patient is under general anesthesia and placed in a supine position. A Foley catheter is inserted into the bladder on the sterile field to allow bladder filling and emptying during the procedure. The first 8 mm port for the 30-degree optic is positioned transumbilically using the Hasson technique. Pneumoperitoneum is achieved with a pressure of 10–12 mmHg. Two accessory ports are placed 7–9 cm apart from the camera port along the mid-clavicular line. A 5 mm accessory port for the bed side is positioned at the site opposite the renal pathology. A Trendelenburg position is achieved to provide better exposure of the pelvic organs before da Vinci robot docking is performed. In fact, once the robotic arms are engaged, the patient cannot be moved without disengaging the robotic device [6,7,8].

After the initial endoscope arm is docked to its port, the endoscope is inserted, and the targeting is started. The remaining arms are then docked to their respective ports. Instruments should be always introduced under vision. The surgical procedure starts with an incision in the peritoneum above the posterior bladder wall on the affected side, below the bifurcation of the iliac vessels, to identify the ureter. The ureter is mobilized to the level of vas deferens or the uterine artery according to patient sex. The nerve plexus is visualized once the ureter has been mobilized and retracted medially. It is important to avoid excessive electrocauterization while dissecting the ureter to prevent injury of the nerve plexus. Using a cotton tape around the ureter can be beneficial to prevent direct robotic manipulation during ureteral dissection. The bladder is suspended to the abdominal wall using a transabdominal suture and filled with saline. A 2.5–3 cm incision is made in the detrusor muscle down to the level of the mucosa. The detrusor muscle is then dissected laterally away from the mucosa, creating muscular flaps that will be used to construct the detrusor tunnel. It is essential to construct a detrusor tunnel with a minimum tunnel length-to-ureter diameter ratio of 4:1 and to sufficiently mobilize the detrusor flaps laterally to prevent tension during tunnel closure. The flaps are then wrapped around the ureter and secured with 4–0 polyglactin sutures. It is crucial to ensure the ureter is neither constricted nor kinked during the procedure. The anastomosis can be completed with either interrupted or continuous sutures, with a preference for using a monofilament suture. The peritoneum is then closed. The working ports must be removed under direct vision. Usually, the bladder catheter is maintained for 24 h in unilateral cases and 48 h in bilateral cases. The child must urinate before discharge. No JJ stent or drain is used when the ureter is not dismembered [6,7,8].

## 2. Materials and Methods

For this study, an electronic literature search of PubMed was performed. Using Boolean operators AND/OR, all possible combinations of the following search terms were used: “robot-assisted extravesical ureteral reimplantation”, “REVUR”, “robot-assisted laparoscopic ureteral reimplantation”, “RALUR”, “robot-assisted laparoscopic extravesical ureteral reimplantation”, “RALUR-EV”, and “pediatrics”. The inclusion criteria were articles published in PubMed limited to the English language and related only to pediatrics (patients under 18 years of age). The exclusion criteria were articles for which full texts was not available, those with content redundancy, and those not written in English. Case reports, video banks, commentaries, editorials, short notes and letters to the editor, off-topic articles, and duplicates were excluded. All studies examining robotic ureteral reimplantation for conditions other than vesicoureteral reflux, such as obstructive megaureter, were excluded from this review. From the articles retrieved in the initial search, additional references were identified through a manual search of the cited references. 

## 3. Results

**Figure 1:**
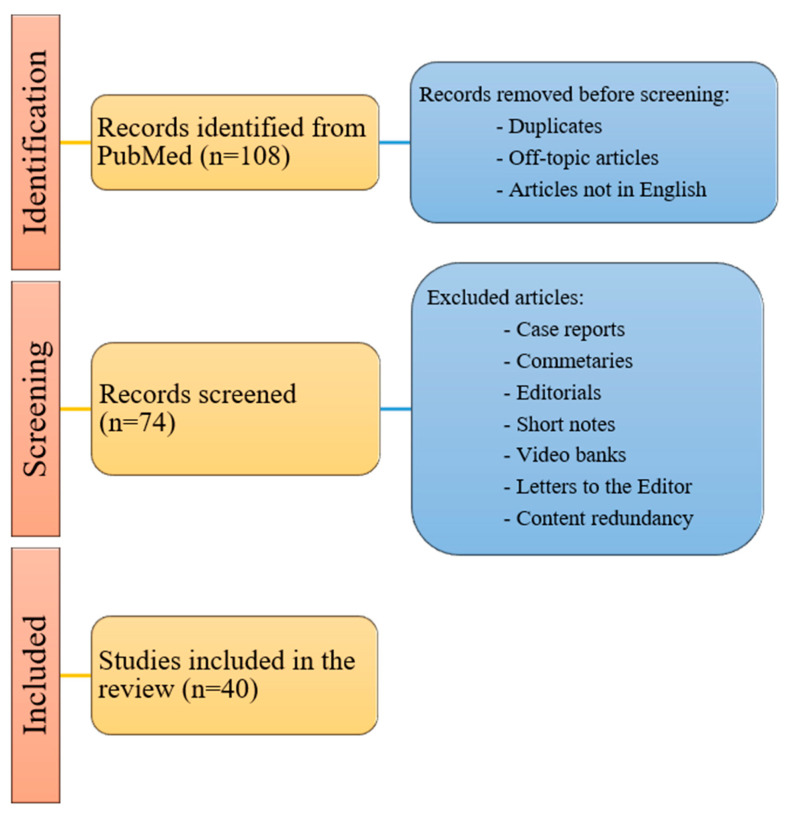
PRISMA flow chart of literature search strategy.

A total of 40 articles published from 2004 to June 2024 were obtained. Figure 1 reports the PRISMA flow chart of the literature selection process.

### 3.1. Robot-Assisted Ureteral Reimplantation: Intravesical vs. Extravesical Approach

This review does not aim to delve into the specifics of robotic intravesical ureteral reimplantation. Rather, this paragraph will compare the intravesical and extravesical approaches two techniques and highlight the reasons found in the literature that make robot-assisted extravesical reimplantation (REVUR) the most adopted method. The most used technique for robot-assisted ureteral reimplantation is derived from the extravesical, transperitoneal Lich–Gregoir procedure [9,10], which was first introduced by Peters in 2004 [1]. Gundeti et al. [11] reported that the intravesical approach has several technical difficulties. These include maintaining pneumovesicum, trocar placement issues, postoperative leaks from the trocar site, and challenges in maneuvering the instruments within small capacity bladders [11]. For this reason, there are few studies in the literature that report the outcomes of this approach. Moreover, these studies involve a small number of patients [12,13,14]. They are summarized in Table 1.

Extravesical ureteral reimplantation allows the bladder to maintain a normal anatomy, which can be beneficial for the child if further surgical procedures are needed in the future [15]. Additionally, the bladder is not opened during this operation, potentially resulting in less postoperative bleeding and fewer bladder spasms for the patient [15].

Furthermore, it tends to result in lower morbidity, with less postoperative bleeding and fewer bladder spasms compared to an intravesical procedure [15].

In 2004, Peters et al. [1] reported on an initial series of 24 children who underwent robot-assisted laparoscopic ureteral reimplantation. Since then, larger series have been reported on with satisfying results [7,8,16,17,18,19,20].

The REVUR technique allowed Chalmers et al. [21] to achieve complete vesicoureteral reflux resolution in 20 ureters (90.9%), downgrading in one ureter, and unchanged persistent reflux in one ureter.

In 2014 Hayashi et al. [22] showed a postoperative reflux resolution in 14 of the 15 ureters (success rate 93.3%) confirmed with a postoperative voiding cystourethrography. In the remaining three patients, which involved reflux in a unilateral ureter, the grade of the reflux decreased from III to I [22].

Boysen et al. [23] reported an overall radiographic success rate of 93.8%, with a rate of 94.1% specifically among children with VUR grades III and V. The variability in published outcomes is challenging to attribute to specific factors. However, differences in patient characteristics (such as age, the presence of BBD, and preoperative VUR grade) as well as variations in surgical techniques may contribute to these discrepancies [23]. In addition, surgeons appreciate the ergonomics and the better visualization enabled by robotic technology. Results from studies evaluating the outcomes of extravesical approach are summarized in Table 2.

### 3.2. Open Ureteral Reimplantation vs. Robot-Assisted Reimplantation

For many years, open ureteral reimplantation (OUR) was regarded as the gold standard treatment for patients with VUR requiring surgical intervention and was the most commonly used surgical approach for ureteral reimplantation [25,26,27]. Conversely, REVUR is increasingly being adopted as the preferred treatment for high-grade VUR in numerous centers around the world [11]. OUR is a very effective procedure, achieving correction rates of 95 to 99 percent, regardless of the severity of VUR [27,28,29]. 

Open procedures are based on the fundamental technique outlined by Politano and Leadbetter, and include the intravesical approach (along with its variants such as Cohen) and the extravesical approach (Lich–Gregoir), which do not require opening the bladder.

Over the past 20 years, robot-assisted extravesical ureteral reimplantation using the Lich–Gregoir technique has become increasingly accepted [30]. This approach reduces the morbidity linked to traditional open intravesical reimplantation, resulting in reduced incidences of postoperative hematuria and bladder spasms, as well as shorter hospitalizations [22,30]. These results are confirmed by literature retrospective scientific studies on the comparison between robot-assisted ureteral reimplantation and the open approach, focused on estimated blood loss and length of hospital stay [31,32].

Harel et al. [33] found that robotic reimplantation resulted in lower postoperative narcotic use and less intense pain compared to open intravesical repair, decreasing the postoperative morbidity.

A review of the international literature reveals that the success rate of REVUR is comparable to that of open reimplantation [12,34]. Marchini et al. [12] demonstrated that robot-assisted laparoscopic ureteral reimplantation offers similar success rates compared to open ureteral reimplantation. Moreover, it provides shorter hospital stays and bladder catheterization time compared to intravesical techniques [12].

All the papers that compare the open vs. the robot-assisted approach reported a longer operative time and a higher cost for REVUR. Sforza et al., in 2024 [35], reported a median operative time of 100 min for the open ureteral approach and 120 for the robot-assisted technique.

While postoperative pain medication usage was significantly lower in REVUR [12,34,36,37,38].

De novo hydronephrosis can occur in up to 30% of cases following extravesical robot-assisted laparoscopic ureteral reimplantation. This condition behaves similarly to that seen after open ureteral reimplantation and resolves on its own in most cases [37]. This is further supported by the study conducted by Babajide et al. [39]. They reported a de novo occurrence of hydronephrosis in 30.8% of patients who underwent OUR and in 27.6% of patients who underwent robot-assisted ureteral reimplantation [39]. Furthermore, although spontaneous resolution was possible in both cases, their study indicates a higher likelihood of resolution at any point during follow-up in the REVUR group compared to the OUR group [39]. In these cases, Mittal et al. [40] recommend maintaining long-term ultrasound follow-up.

Another aspect to analyze is the cost to the hospital. The total hospital charges for robot-assisted ureteral reimplantation were generally higher than those for OUR, largely due to the increased costs associated with robotic equipment and longer operative times [41]. Despite higher upfront charges, the shorter hospital stays and quicker recovery times with robot-assisted technique might lead to cost benefits in the long term, such as reduced postoperative care needs [41]. The results of the open approach are reported in Table 3.

### 3.3. Laparoscopic Reimplantation vs. Robot-Assisted Reimplantation

Conventional laparoscopic ureteral reimplantation has been an option, but it is challenging due to the difficulties in suturing and knot tying [38,42]. Several papers have reported resolution rates of 87–100% using this technique [30,43,44,45,46].

In 2016, Esposito et al. [30] published a study that compared the endoscopic, laparoscopic, and open surgical approaches for VUR. They found that both laparoscopic extravesical ureteral reimplantation (LEVUR) and open Cohen techniques had higher success rates than the subureteric transurethral injection (STING) procedure. However, both STING and LEVUR were associated with shorter and less painful hospital stays compared to the open approach [30]. Different authors in fact have highlighted the safety and effectiveness of conventional laparoscopic ureteral reimplantation using an extravesical approach [47,48,49].

The primary advantages of laparoscopic extravesical ureteral reimplantation (LEVUR) over robot-assisted extravesical ureteral reimplantation (REVUR) are relatively limited but still noteworthy. First, LEVUR utilizes smaller trocars, with a diameter of 3 mm compared to the 8 mm trocars used in REVUR, resulting in less invasive entry points, potentially minimizing tissue trauma and postoperative discomfort. Second, the overall cost of the procedure is lower for LEVUR. While these two benefits may seem modest, they can have a meaningful impact on patient outcomes, especially in pediatric populations, where minimizing invasiveness and reducing costs are important factors in surgical decision-making.

However, LEVUR remains highly challenging and technically demanding. This is also evident in the variability of the learning curve. Lopez et al. [43] believe that this procedure is technically feasible and can be effectively learned by young surgeons undergoing laparoscopic and urologic training. Based on their experience, they observed that the learning curve stabilized after 6 to 7 cases. Additionally, operative times showed a significant reduction after the first 6 cases [43]. Furthermore, as reported by Riquelme et al. in their case series, postoperative complications are more common during the initial procedures and decrease as the number of cases increases and the learning curve flattens [50].

Robot-assisted ureteral reimplantation has the potential to overcome the limitations of the laparoscopic approach, providing enhanced precision and ease in terms of performing complex tasks [48]. For example, in the laparoscopic approach, precision is limited by the surgeon’s manual movements and 2D visualization. Hand tremor can affect precision, especially in delicate procedures. Furthermore, viewing angles are limited by the position and orientation of laparoscopic instruments. The suturing precision is limited, with greater difficulty in terms of controlling suturing and handling delicate tissues, while robotics offers increased precision in suturing and fine movements due to advanced robotic tools. These advantages lead to fewer complications related to technique.

The operative time (OT) of robot-assisted ureteral reimplantation is consistently shorter than for laparoscopic procedures across all studies, due to reduced suturing time and lower surgeon fatigue [38]. The advantages of the robotic technique include enhanced three-dimensional visualization, precision, instrument flexibility, dexterity, and ease in pelvic surgeries [38]. Furthermore, the surgeon has better ergonomics, working seated using robotic controls. For all these reasons and the shorter learning curve [51], REVUR is going to be the minimally invasive treatment of choice.

Esposito et al. [52] recommended opting for REVUR in cases of bilateral and/or high-grade reflux with megaureter, while in cases of unilateral reflux the results of the laparoscopic Lich–Gregoire procedure and REVUR are the same [17], allowing the surgeon to choose between the two techniques based on the different ergonomics preferences. A summary of studies evaluating LEVUR’s outcome is reported in Table 4.

### 3.4. REVUR for Bilateral VUR and Complex Anatomy

While is well demonstrated in the international literature that REVUR is a safe and feasible technique with excellent results for unilateral reimplantation, it is challenging to determine if the same benefits can be achieved for bilateral reimplantation in patients with more complex underlying renal conditions.

Older children with complex anatomy or those who have had a failed injection or open reimplantation can particularly benefit from robot-assisted ureteral reimplantation, as the robotic approach enhances exposure. REVUR can be performed unilaterally or bilaterally; however, caution is recommended in bilateral cases due to the risk of transient urinary retention [22]. 

It is well known in fact that with conventional approach for bilateral ureteral reimplantation, there is a high risk of postoperative voiding complications secondary to visualization of the pelvic plexus [53].

Some authors have investigated whether REVUR is safe and effective for both simple and complex ureteral anatomies. Casale et al. [54] reported the outcomes of 41 patients who underwent robot-assisted extravesical reimplantation for bilateral VUR. During REVUR procedure they provided a careful identification of the pelvic plexus to prevent injury and facilitate ureteral mobilization. Results showed that all patients were able to void spontaneously, with minimal to no postvoid residual observed on bladder scans, averaging 13 mL. Uroflow assessments for all toilet-trained patients remained consistent with preoperative studies [54]. Their results were confirmed in a prospective long-term study that they published in 2012 [55].

Herz et al. [56] reported a 91.7% success rate for unilateral robot-assisted ureteral reimplantation, but only a 77.8% success rate for ureters (72.2% of children) in bilateral cases. This study found higher complication rates (including ureteral obstruction, readmission, and urinary retention) for bilateral cases, and a nerve-sparing technique was not used.

Esposito et al. [57] reported a comparative analysis between simple and complex anatomy that showed that REVUR was faster in simple cases in both unilateral and bilateral repair and postoperative retention was more frequent in simple cases and in patients with pre-operative bowel and bladder dysfunction.

REVUR was safe and effective for the management of VUR in both simple and complex ureter anatomy. Complex REVUR required slightly longer operative times, without significant differences in postoperative morbidity and success rates [57].

As for the nerve sparing, based upon Esposito et al. [57] findings, the nerve sparing technique is useful to prevent injury to the pelvic plexus but does not eliminate at all the risk of postoperative urinary retention. Results are summarized in Table 5.

### 3.5. REVUR as II Line after Endoscopic Injection Failure

Currently, the first line approach seems to be endoscopic injection (EI). EI reported satisfactory outcomes with resolution rate ranging from 69 to 100% [58]. In case of recurrence, it is debated which is the best second line of treatment.

Comez et al. [59] found that 14 patients who had undergone previous STING surgery needed considerably more time for both total operation and console use. This implies that performing STING surgery for reflux is more difficult when a bulking agent and inflammatory changes are involved [59].

Janssen et al. showed that REVUR can be a safe salvage option in patients who have failed EI for VUR [60]. A total of 17 REVUR procedures were reviewed in 16 patients, 7 of them underwent two prior failed EIs. Seven of them underwent two EI treatments prior to REVUR. Among those who underwent postoperative imaging, they reported a success rate of 86% for radiographic outcomes and 88% for clinical outcomes [61].

## 4. Discussion

Robot-assisted extravesical ureteral reimplantation is a safe and minimally invasive method that has gained popularity in pediatric urology over the past few decades [62]. Robotic surgery offers several advantages over conventional laparoscopy, making it particularly suitable for reconstructive procedures involving delicate structures like the ureter. This approach provides enhanced access and visualization of the ureter at every level.

It is important to remember that robot-assisted reimplantation is a relatively new surgery and, consequently, the literature on the subject needs to be expanded, especially when compared to OUR, which has over 50 years of literature.

Open ureteral reimplantation is generally regarded as the preferred method for treating vesicoureteral reflux due to its historically high success rates, which range between 93%, 95%, and 100% [33,62,63].

LEVUR has shown promising outcomes (range between 87 and 100%) [30,43,44,45,46]; however, it has not achieved the same level of popularity as OUR due to its technical challenges.

Despite these success rates, robot-assisted reimplantation has gained significant popularity, becoming the second most common robotic procedure after pyeloplasty in pediatric population [64].

Advantages of the robotic approach compared to the open approach include enhanced visualization through magnification, better intraperitoneal identification of the ureters and bladder, improved cosmetic outcomes for the patient, and quicker recovery during the early postoperative period, especially for extravesical approach.

As for the costs, Kurtz et al. [65] provided in their article a comprehensive analysis of the financial and clinical outcomes associated with robotic versus open techniques for pediatric ureteral reimplantation, highlighting that the higher initial costs of robotic surgery could be balanced by potential long-term benefits in patient recovery and institutional efficiency. However, it should be emphasized that the limitations of this study may include the use of a nationwide sample and the level of experience and expertise of the surgical team. Differences in skill levels and familiarity with robotic technology among institutions could affect the study’s findings.

The varying success and complication rates associated with robot-assisted laparoscopic surgery likely reflect its recent introduction into pediatric urology. We believe that this variation in success rates may be due to several factors such as surgeon’s experience and learning curve, differences in technique, appropriate patient selection, and the severity of VUR. The surgeon plays an important role and must know the robotic technology and the robot-assisted reimplantation techniques to avoid complications due to lack of knowledge, practice, and expertise. In fact, the learning curve for robotic ureteral reimplantation is steep, indicating that mastering this procedure requires significant time and effort. But this is nothing compared to the challenges of performing traditional laparoscopic ureteral reimplantation [66].

Casale et al. [54] reported in 2008 a flattening of the learning curve after five cases for robot-assisted ureteral reimplantation, while the learning curve for extravesical and vesicoscopic laparoscopic approaches plateaued after approximately five to seven cases [64].

Many seasoned pediatric urologists lack formal training in this technology, which hampers their ability to adapt quickly [67]. However, newer fellowship graduates are overcoming the steep learning curve by receiving specialized training during their residencies and fellowships [66].

Compared to traditional laparoscopy, robotic surgery has a quicker learning curve and proves particularly beneficial in pelvic reconstructive procedures, offering excellent postoperative outcomes and few complications [11].

Therefore, there is a need across the discipline to standardize the current REVUR technique to maximize surgical success.

The international literature extensively describes how robotic surgery has not only significantly improved precision and outcomes in traditional procedures such as ureteral reimplantation but has also broadened its applications into new delicate areas such as uro-oncology [68,69,70].

However, these benefits come with significant costs such as the purchase of the robot itself, maintenance expenses, and the use of expensive instruments, many of which are disposable. Nevertheless, as previously noted, if we consider that a reduced hospital stays, shorter convalescence, fewer postoperative complications, and a decrease in the need for re-interventions can offset the costs, then the investment may be justified. Furthermore, with the growth of the robotic market and the emergence of more cost-effective alternatives, there is a real prospect for global access to minimally invasive surgery [71].

Robot-assisted laparoscopic ureteral reimplantation is a minimally invasive technique that is practical, safe, and associated with reduced morbidity. It provides effective results and a more esthetically pleasing approach to ureteric reconstruction. The majority of patients are highly satisfied with this method. 

## 5. Conclusions

While the steep learning curve might be a deterrent to the adoption of this technique, thanks to modern and increasingly widespread training programs, and by following clear and simple steps during the surgical procedure with the right precautions—such as the proper use of electrocautery—it is possible to state that REVUR is a safe, easily executable technique with excellent outcomes.

Based on the literature, REVUR is emerging as the technique of the future, as evidenced by the increasing number of robot-assisted reimplantation procedures. Conversely, the open and laparoscopic approaches are being used less frequently. Currently, REVUR is a feasible and valuable choice for VUR treatment, especially for high-grade, previous failure treatment, and bilateral ureteral reimplantation. It should be noted that the high costs and the need for specialized centers may limit the widespread adoption of this technique.

Currently, REVUR remains a technique that should be performed in specialized centers by well-trained surgeons in order to obtain the best outcomes for the patients. For this reason, it is advisable to refer selected patients to specialized centers to offer children the best surgical technique available based on their specific needs. With the presence of numerous centers offering training in robotic surgery, the hope for the future is to achieve a reduction in the production costs of the robot, the establishment of new specialized centers and the introduction of more child-friendly instruments.

## Figures and Tables

**Table 1:** Results from studies evaluating outcomes of intravesical approach.

Study	Patients (n)	Success Rate (%) ^1^	Hospitalization Rate (Range Days)	Complications Rate (%)	Conversion Rate (%)
Peters (2005) [13]	6	83.3	2–4	17	0
Marchini (2011) [12]	19	92.2	NA ^2^	52	0
Chan (2012) [14]	3	100	2–7	0	0

^1^ Confirmed by postoperative voiding cystourethrogram (VCUG); ^2^ NA: not available.

**Table 2:** Results from studies evaluating outcomes of extravesical approach.

Study	Patients (n)	Success Rate (%) ^1^	Hospitalization Stays (Days)	Complications Rate (%)	Conversion Rate (%)
Marchini (2011) [12]	20	95	1.7	10	0
Chalmers (2012) [21]	16	87.5	1.3	0	0
Akhavan (2014) [7]	50	92.3	2.0	10	0
Hayashi (2014) [22]	9	93.3	7.4	0	0
Boysen (2018) [23]	144	91.4	1.5	6.3 ^2^	0.69

^1^ Confirmed by postoperative voiding cystourethrogram (VCUG); ^2^ Clavien–Dindo grades 3 (5.6%) and 2 (0.7%) [24].

**Table 3:** Results from studies evaluating outcomes of open approach.

Study	Patients (n)	Mean Operative Time (min)	Success Rate (%)	Hospitalization Stays (Range Days)	Complications Rate (%)
Kennelly (1995) [27]	91	180	98.3	5.6 (median)	18
Hubert (2012) [28]	965	NA	93.9	NA	13
Harel (2015) [33]	11	188	100	1–>2	18
Sforza (2024) [35]	68	100	94	3–6	7.4 ^1^

^1^ Clavien–Dindo grade 1 [24].

**Table 4:** Results from studies evaluating outcomes of laparoscopic ureteral reimplantation.

Study	Patients (n)	Ureters (n)	Mean OT ^1^ (min)	Success Rate (%) ^2^	Mean HS ^3^ (h.)	Complications Rate (%)	Conversion Rate (%)
Lopez (2010) [43]	30	43	97	100	24	7	0
Castillo (2013) [44]	42	50	74	100	96	0	0
Riquelme (2013) [50]	81	95	109.3	95.8	38.4	2.1	0
Soulier (2017) [45]	117	159	112	98.3	25.3	6.8	0
Badawy (2017) [46]	17	NA	90	100	48	17	0

^1^ OT: operative time; ^2^ confirmed by postoperative voiding cystourethrogram (VCUG); ^3^ HS: hospital stays.

**Table 5:** Results from studies evaluating bilateral VUR, complex anatomy with or without nerve-sparing technique.

Study	Patients (n)	Operative Time (h.)	Success Rate (%) ^2^	Mean Hospital Stays (h.)	Complications Rate (%)	Nerve-Sparing Technique (Y/N)
Casale (2008) [55]	41	2.33 ^1^	97.6	26.1	0	Y
Kasturi (2012) [56]	150	1.8 ^1^	99.3	22.1	0	Y
Herz (2016) [57]	54	NA	84.7	39.36	11.1	N
Esposito (2023) [58]	57	3.31 ^3^	96.5	52.8	21 ^4^	Y

^1^ Including cystoscopy; ^2^ confirmed by postoperative voiding cystourethrogram (VCUG); ^3^ 2.35 unilateral and 3.31 bilateral; ^4^ Clavien Dindo grade 1–2 [24].

## Data Availability

Not applicable.
